# A review of the framework and strategy for disability and rehabilitation services in South Africa

**DOI:** 10.4102/ajod.v11i0.893

**Published:** 2022-12-15

**Authors:** Naeema A.R. Hussein El Kout, Sonti Pilusa, Khetsiwe Dlamini Masuku

**Affiliations:** 1 Department of Physiotherapy, Faculty of Health Sciences, University of the Witwatersrand, Johannesburg, South Africa; 2 Department of Speech Pathology and Audiology, Faculty of Health Sciences, University of the Witwatersrand, Johannesburg, South Africa

**Keywords:** rehabilitation, access, framework and strategy for disability of South Africa, policy, actors, processes, context, content

## Abstract

**Background:**

Rehabilitation is imperative for the successful integration of persons with disabilities into their social environments. The Framework and strategy for disability and rehabilitation services (FSDR) in South Africa, 2015-2020.was developed to strengthen access to rehabilitation services and ensure the inclusion of persons with disabilities in all aspects of community life. Despite the FSDR being commissioned, access to rehabilitation is a challenge for persons with disabilities and further compounded in rural communities.

**Objective:**

The study aimed to describe the barriers and facilitators that influenced the process of development, implementation and monitoring of the FSDR.

**Method:**

This qualitative study employed a single case study design. Data was collected through document analysis and in-depth interviews utilising the Walt & Gilson policy analysis framework that outlines the context, content, actors and process of policy development and implementation. In-depth interviews were conducted with twelve key informants (N=12) who were selected purposively for the study. Data obtained from the in-depth interviews were analysed using inductive thematic analysis.

**Results:**

We found many factors that influenced the implementation of the framework. Actor dynamics, insufficient resources, the rushed process, poor record-keeping, inappropriate leadership, negative attitudes of staff members and the insufficient monitoring impeded the successful implementation of the framework. While positive attitude, mentorship and support amongst the task team facilitated the implementation process, albeit with challenges.

**Conclusion:**

There is a need to address implementation gaps so that the FSDR is responsive to the current rehabilitation needs of persons with disabilities in South Africa.

**Contribution:**

This study may inform future disability policy, and can be used as a tool to advocate for the rights for persons with disabilities

## Introduction

Approximately 80% of the global population of persons with disabilities is in developing countries (United Nations 2019). To date, statistics on the prevalence of disability in South Africa are inconsistent. For example, in 2016, the national prevalence of disability was estimated to be 7.5% (Statistics South Africa 2014), a percentage that does not include children under the age of 5 years old and people presenting with psychological and neurological conditions (Sherry 2014). On the contrary, the World Health Organization (2011), using data obtained from the World Health Survey between 2002 and 2004, reported a disability prevalence of 15% globally and 24.2% in lower-income countries. The quadruple burden of disease influences the prevalence of disability in South Africa, particularly maternal and child health, HIV/AIDS, trauma and violence and non-communicable diseases (Kietrys et al., 2019; Maredza & Chola 2016; Mayosi et al. 2009). In low- and middle-income countries (LMICs) such as South Africa, disability and poverty are also often intertwined (Mitra, Posarac & Vick 2013), with persons with disabilities being most likely to be poor because of exclusion from educational, employment and economic opportunities (Pinilla-Roncancio 2015).

Persons with disabilities experience significant health challenges and healthcare needs (Krahn, Walker & Coorea-De-Araujo 2015). Persons with disabilities tend to experience secondary complications because of their primary disability (Maart, Amosun & Jelsma 2019). Persons with disabilities also require rehabilitation to realise their full health and functioning (Sherry 2014). Rehabilitation has been defined as a grouping of interventions that aim to reduce disability and improve the functionality of individuals (Philpott, Mclaren & Rule 2020). As such, rehabilitation care should form a significant part of primary healthcare (Sherry 2014). However, access to healthcare, including rehabilitation, is limited for persons with disabilities (Bright, Wallace & Kuper 2018).

Persons with disabilities struggle to access healthcare services because of many barriers. Some of the barriers include transportation challenges and finances related to transportation costs, geographical location of healthcare facilities, physical access at the healthcare facilities, long queues and waiting times, poor access to communication and health information, attitudes of healthcare professionals, availability of medicine and equipment and inadequate referral systems (Braathen et al. 2016; Moodley & Ross 2015; Sherry 2014; Van Rooy et al. 2012; Visagie & Schneider 2014). Service barriers specific to rehabilitation services include the shortage of rehabilitation staff workers (Maart & Jelsma 2013), which usually encompasses physiotherapists, occupational therapists, speech-language therapists and audiologists (Visagie et al. 2017). Other challenges include an inadequate budget allocation for services and assistive devices, length of hospital stay, poor intersectoral collaboration and language barriers (Maart & Jelsma 2013; Ntamo, Buso & Longo-Mbenza 2013). These barriers are worse in rural South African contexts (Vergunst et al. 2017). Subsequently, not meeting the health and rehabilitation needs of persons with disabilities may lead to poorer health, decreased quality of life, increased re-hospitalisation, and ultimately perpetuate their discrimination and exclusion from participation in society (The South African Human Rights Commission 2019).

Access to healthcare is a human right for all, as articulated in Articles 25 and 26 of the Convention on the Rights of Persons with Disabilities (CRPDs) (United Nations 2006). South Africa ratified the CRPD in 2007 (Hussey, MacLachlan & Mji 2017). After the ratification of the CRPD, South Africa developed the White Paper on the Rights Of Persons With Disabilities in 2015 (Kamga 2016) and the framework and strategy for disability and rehabilitation services in South Africa, 2015–2020 (FSDR). The development of the FSDR commenced in 2013, and its implementation was intended for 2015–2020 because of the COVID-19 pandemic, it was extended to 2022 (Sherry 2014). Implementation was aimed at national and provincial rehabilitation managers, rehabilitation professionals as well as community health workers. However, there is poor awareness of the FSDR amongst the stakeholders, which has resulted in little known information on the implementation process and outcomes. The FSDR is based on primary healthcare re-engineering and pillared on the principles of community-based rehabilitation (CBR), which is arguably the golden standard for rehabilitation in both developed and developing countries because of its emphasis on inclusion (Grandisson, Hebert & Thibeault 2014). Despite the existence of these progressive disability rights policies and their good intentions, disability scholars in South Africa continue to lament the barriers that persons with disabilities experience when they access healthcare and rehabilitation services (Moodely & Ross 2015; Sherry 2014), with the magnitude and consequences of these barriers differing with each specific context (World Health Organization 2011).

## Problem statement

Despite the increasing prevalence of disability in South Africa and globally, disability rights are not upheld. A tool to address this is public health policy. The year 2020 marked 5 years since the inception of the FSDR. Prior to this study, the FSDR had never been reviewed. This study aimed to describe the FSDR processes (actors, content and context) and the barriers and facilitators that influenced the policy processes (development, implementation and monitoring) of the FSDR of South Africa.

## Conceptual framework

The authors utilised the Walt and Gilson (1994) triangle framework for policy analysis, which provides a holistic and systematic approach to policy analysis from the perspective of the content, context, actors and policy processes (Blaauw, Ditlopo & Rispel 2014; Walt & Gilson 1994). This study focused on the process aspect, namely development, implementation and monitoring and evaluation and how these processes were influenced by barriers and facilitators. Process pertains to the descriptions of how policy reforms were identified, formulated, implemented and monitored as well as the various stages involved (Buse, Mays & Walt 2005). The advantage of using this framework is that it was intended for policy analysis in developing countries (Walt et al. 2008).

The Walt and Gilson analytical framework was developed specifically for healthcare and has been widely used on a substantial number of health issues (Gilson & Raphaely 2008). Most importantly, the analytical framework is based on a political economy perspective considering how content, context, actors and processes interconnect to influence policymaking (Walt et al. 2008). Even though there are several policy reforms on disability and access to healthcare, the FSDR focuses specifically on rehabilitation and the interface between rehabilitation, CBR and primary healthcare re-engineering. Policy reforms are generally political in nature (Buse et al. 2005). As such, policy analysis using a structured framework such as the Walt and Gilson framework is required.

## Research methods and design

### Research design

To achieve the aim of this qualitative research, a single case study research design was employed to ascertain the actors, content, context and processes of the FSDR. A single case study design is a valuable tool to explore complex issues and relationships within a real-life context such as the processes’ barriers and facilitators (Tetnowski 2015; Yin 1999).

### Study population

The study involved 12 key informants (Table 1) who were selected through purposive and snowball sampling for the study. Purposive sampling was deemed appropriate because it allowed the authors to identify and select participants whose experiences can answer the research question (Tongco 2007) and provide detailed and rich information related and pertinent to the phenomenon of interest (Palinkas et al. 2015). Purposively selected participants followed the study inclusion criteria that referred to individuals who were involved in the development, implementation and monitoring process of the FSDR namely, rehabilitation services managers and therapeutic sciences professionals as well as academia, professional organisations for therapeutic sciences and disability persons organisations. From these primary participants, secondary participants were identified through snowball sampling which includes a member of professional organisations for rehabilitation, a member of the district rehabilitation division and two individuals from disability persons organisations, all of whom were involved in development and implementation of the FSDR.

**TABLE 1:** Demographic information of key informants.

Demographic information	*n*	%
**Gender**		
Female	10	83.33
Male	2	16.67
**Age**		
25–35	4	33.33
36–45	3	25.00
46–55	2	16.67
56–65	2	16.67
66–75	1	8.33
**Level of education**		
PhD (all conducted in field of rehabilitation and therapeutic sciences)	3	25.00
Masters	1	8.33
Bachelor’s degree	11	91.67
Diploma	1	8.33
**Sector**		
Professional association and private sector	3	25.00
Public sector	7	58.33
Academia	1	8.33
Non-governmental organisation	1	8.33

PhD, Doctor of Philosophy.

The 12 key informants represented four stakeholder groups who are based in the Johannesburg Metropolitan District: Gauteng Department of Health (*n* = 7), Professional associations (*n* = 3), Academics (*n* = 1) and non-governmental organisations (*n* = 1). The demographic data of the participants are represented in Table 1.

The key informants were professionals registered with the Health Professionals Council of South Africa (HPCSA) who work in academia (one participant), in the non-government organisation sector (one participant) and in both the public (seven participants) and private sectors (three participants). The participants were in these positions since the inception of the FSDR. A total of 10 females and two males participated in the study. All participants had work experience in the field of rehabilitation and disability, either clinical experience, academic research or in management positions associated with these fields. The participants’ involvement in the FSDR ranged from development to implementation to monitoring, with some individuals involved in all three processes. Participant one was an academic with over 30 years of rehabilitation experience and was involved in only development, despite their involvement in a disability person’s organisation. Participant two, was from the public sector, in an influential managerial position associated with rehabilitation and was involved in all three processes. Participants three to six, were rehabilitation professionals on the ground, who were involved in development and implementation. Participants seven and eight, held management positions within the public sector in rehabilitation in the district. The remaining participants were clinical rehabilitation professionals at ground level who were excluded from development but involved in the implementation process.

### Data collection

The FSDR is the only policy that speaks to disability and rehabilitation in South Africa. The FSDR was searched and retrieved online. A document review of the FSDR was conducted using review guide (Appendix 1) that was developed by the researchers informed by the Walt and Gilson (1994) Health Policy Analysis Triangle Framework. The purpose of this review is to identify the key role players involved in the processes, as well as the content, context and processes of the FSDR.

Semi-structured interviews were conducted using an interview guide (Appendix 2) developed by the authors. The interview covered topics that would answer the research question, namely actors, context, content and the processes of the FSDR and access to healthcare. Prior to the main study, a pilot study was conducted to establish the relevance and appropriateness of the interview guide questions and familiarise the researcher with the research process (Van Teijlngen & Hundley 2002). All 12 interviews were audio-recorded using a mobile phone recorder and the recordings were stored in a secured folder. Interviews were conducted until data saturation was reached when repetition occurred in the interviews and no new themes or points of interest arose (Guest, Bunce & Johnson 2006).

### Data analysis

The researchers read the document line by line independently and systematically coded the document using MAXQDA version 2018.2 (Berlin, Germany): a software programme for qualitative data analysis. Coding independently was to ensure inter-rater reliability and agreement of the data (Campbell et al. 2013). Thereafter the researchers discussed and compared the coded text segments with the three coders. All the coded segments in the FSDR document were categorised into content, actors and context. Discussion among the researchers on the coding process and categories were performed on a regular basis.

The interviews were transcribed verbatim and rechecked to ensure correct transcription (Braun & Clarke 2006). The first author engaged with the transcripts for familiarity by reading and reading them and correcting grammar or typing errors. Memos were added to the transcripts as they were read. The MAXQDA version 2018.2 was used for data analysis.

Inductive thematic analysis was used as it is deemed an accessible and flexible approach to the analysis of qualitative data (Braun & Clarke 2006). The (Author(s) in press) searched for broader memos in the transcripts. From this, the memos were sorted into different codes. The codes were categorised into sub-themes and then sorted into the main themes (Erlingsson & Brysiewicz 2017; Hsieh & Shannon 2005). Recoding was redone by the principal researcher. Regular meetings were held by the researcher to discuss the research procedure, preliminary findings and the final study findings.

### Trustworthiness

To ensure trustworthiness and rigour, this study utilised member checking by confirming participants’ responses immediately after each interview to ensure the responses documented and recorded by the researcher correlated with what the participant intended to say (Shenton 2004). Transferability was achieved utilising detailed descriptions of the study methodology (Shenton 2004). Dependability was achieved through in-depth descriptions of the data collection methods. Confirmability was achieved through the reflective commentary from the reflective journal with observations on verbal cues and body language, as well as in-depth descriptions of the methodologies used.

### Ethical considerations

Ethical clearance was granted by the Medical Human Ethics Research Committee of the University of the Witwatersrand (M190438). The research protocol was registered on the National Health Research Database. Permission was granted by the Gauteng Department of Health research committee (GP_201905_45). Study participants were approached for participation, the aim of the study was explained to the participants in an information sheet and written informed consent was obtained. A separate consent for the interviews and a separate form for the audio recordings were completed. Each participant remained anonymous using identifier codes and the concealment of participant’s specific workplaces to maintain confidentiality.

## Results

The findings of this article will describe the actors, content, context and processes of the FSDR and the barriers and facilitators that influenced the process of development, implementation and monitoring.

### Framework and strategy for disability and rehabilitation services content

The content of the FSDR covered the aims, distributional impact and foundational philosophy. The document review revealed that the aim of the FSDR was mainly to define and guide rehabilitation service provision:

‘… to achieve equal access to healthcare services as well as to provide specified rehabilitation services where and when required’ (FSDR document, p. 7)

‘The provision of integrated, comprehensive, appropriate disability and rehabilitation services through effective and equitable resource allocation and inter-sectoral collaboration.’ (FSDR document, p. 11)

The key informants focused more on the allocation of resources and intersectoral collaboration:

‘I feel the FSDR does pull that back together and say firmly we are looking at CBR approach, we are looking at a multidisciplinary rehabilitation team …’ (Participant 4, female, central hospital staff)

‘I think what it does is that it talks to interprofessional and intersectoral collaboration.’ (Participant 2, female, Academia)

‘… So… FSDR also seeks to say how can we structured the human resource aspect.’ (Participant 12, female, specialised hospital)

The FSDR distributional impact was supposed to be realised in all levels of care:

‘This Policy Framework and Strategy for Disability and Rehabilitation services in South Africa outlines comprehensive and integrated disability and rehabilitation services within the broader health and developmental context to facilitate improved access at all levels of healthcare.’ (FSDR document, p. 8)

However, there was mixed feelings from the interviewees. Some participants perceived the FSDR to be effective in terms of improved organisation of rehabilitation systems:

‘I think in some ways it did help us to actually organise our thoughts and give us some motivation or drive to start doing strategic planning you know more formalised and less haphazardly.’ (Participant 8, female, National department of health)

The FSDR also increased awareness of the process of rehabilitation:

‘I think that’s guided us quite a bit as well in terms of understanding the different levels of care provide for the patients and enable them to direct them appropriately and also looking at the referral system between the levels of care and the referrals to the rehab units.’ (Participant 10, female, JHB metropolitan district staff)

On the contrary, some participants were sceptical about the value of FSDR, being framework and not a policy:

‘… It [FSDR] is only a framework and so on, it’s not a full policy that has been accepted and so on. So, in that sense it has less of a value than what a full policy, implemented policy document would be.’ (Participant 7, female, Gauteng department of health rehabilitation)

Some respondents felt that the FSDR lacked substance was not effective and that it had contradictions as explained by the following respondents:

‘… It’s a very scant document. There’s not enough to develop an action plan.’ (Participant 12, female, specialised hospital)

‘There are contradictory things for me in the FSDR, which are contradictory to how people could practice …’ (Participant 2, female, academia)

The guiding philosophy behind the FSDR was CBR as outlined in the document and by the respondents:

‘This strategy focuses on the mandate of the health sector but fully subscribes to the CBR philosophy.’ (FSDR document, p. 13)

‘I feel the FSDR does pull that back together and say firmly we are looking at CBR approach, we are looking at a multidisciplinary rehab team …’ (Participant 4, female, central hospital)

### Context

The context that influenced the development of the FSDR was categorised into structural, cultural, situational and global contexts.

#### Structural context

The need for a new rehabilitation policy that can address the gaps that are presented by the medical model was one of the reasons for developing the FSDR:

‘A medical model resulted in poor access to a comprehensive disability and rehabilitation service especially to persons in rural and disadvantaged areas.’ (FSDR document, p. 10)

‘The gap is that there is nothing in terms of, let’s say, a rehab policy in South Africa.’ (Participant 12, female, specialised hospital)

#### Situational context

Health system inefficiencies such as the lack of access to healthcare, non-integrated services, inadequate resources provision and a lack of rehabilitation indicators necessitated the need for the policy reform:

‘The implementation of disability and rehabilitation services as a vertical programme with little or no scope for integration with priority health programmes, such as Non-Communicable Diseases, Maternal Child and Women’s Health (MCWH), HIV and AIDS.’ (FSDR document, p. 10)

‘Inaccessibility of health services with regard to facility infra-structure, signage and information in an appropriate medium including sign language and Braille.’ (FSDR document, p. 10)

‘It’s like we are uhm these step-sister people, which you call on when you don’t know what to do with anybody.’ (Participant 2, female, academia)

‘What is very clear is that rehab is an afterthought. It’s not an integral part of the health system.’ (Participant 12, female, specialised hospital)

‘So, a process was set in motion then you know to look at that and then to try as much as possible to have then an inclusive approach to disability and rehab services.’ (Participant 9, male, JHB Metropolitan district)

#### Global context

There was international pressure to develop a disability policy to support the United Nations Convention on the Rights of Persons with Disabilities (UNCRPD) as explained in the FSDR:

‘The UN Convention on the Rights of Persons with disability (CRPD) was signed and ratified by the South African government in 2007 and its provisions reflect the obligations of the State. (FSDR document, p. 10) and the interviews I think it had to do with the fact that our government has signed a number of agreements. And rehab internationally is something that is on the agenda, which has to be looked at.’ (Participant 12, female, specialised hospital)

### Actors

The FSDR report stated the importance of involving different stakeholders, which was confirmed in the interviews. There was a range of stakeholders (actors) in the FSDR development, implementation and M&E. The actors included the National Department of Health (rehabilitation manager, clinicians, provincial rehabilitation coordinators and community health workers), consultants, private health groups, traditional healers and NGOs, public sector representatives and professional associations and academia:

‘This Framework and Strategy for Disability and Rehabilitation Services in South Africa 2015 was compiled in consultation with people with disabilities, the Task Team on Disability, professional rehabilitation service providers, academics and other key stakeholders in the field.’ (FSDR document, p. 3)

From the interviews the participants indicated that different stakeholders played different roles throughout the policy process. The rehabilitation manager had a critical role throughout the policy process, to facilitate the development, implementation and oversee the monitoring processes:

‘I became the secretariat for the task team. I was responsible for organising meetings and recording meetings half the meetings I also facilitate. So, I would play both roles of facilitating and secretariat. I had the responsibility to facilitate implementation and essentially the only one involved in M and E.’ (Participant 9, male, JHB Metropolitan district)

Although the clinicians were not involved in the development of the FSDR, they played a critical role in implementing it, as seen in the following quote:

‘We looked at how we could use that (FSDR) in our context, and implement what was recommended in our context …’ (Participant 10, female, JHB Metropolitan district)

Other stakeholders such as the NGOs, public sector representatives and professional associations and academia played a role in the development of the FSDR and no role in the implementation and M&E process:

‘my role was simply to make sure that the rural anomalies and needs and primary healthcare needs and strategies were firmly articulated within the FSDR …’ (Participant 4, female, Central hospital)

‘… [*A*]s part of the task team sort of we had to look at what’s involved in the discussions around developing the document…providing information to the associations after a meeting, get the feedback of what was required and the task that we had to do.’ (Participant 7, female, Gauteng department of health rehabilitation management)

### Policy process: Development, implementation and monitoring and evaluation

The processes of development, implementation and monitoring of the FSDR were faced by both barriers and facilitators as reported by key informants.

#### Development of the framework and strategy for disability and rehabilitation services

A task team in consultation with various stakeholders developed the FSDR. The development of the FSDR was made difficult by poor management in terms of a lack of remuneration for the services rendered:

‘I basically had to work for free for those 3 months – they paid me in the end for I think 23 days – but it took me 3 full months day and night.’ (Participant 5, female, rehabilitation non government organisation)

There was insufficient documentation of the development process as expressed by the following quote:

‘There were no minutes taken…nothing was verbally documented nor was it audio recorded.’ (Participant 4, female, Central hospital)

This lack of documentation was exacerbated by a rushed development process as seen in the response below:

‘They [NDOH] said it (FSDR) had to be developed in a maximum of I think-15 days.’ (Participant 5, female, rehabilitation nongovernment organisation)

Although the development process of the FSDR encountered barriers, there was good teamwork, which facilitated the process. The team was diverse and had a broad range of experience with a positive attitude towards the process:

‘So, we had a broad range of representation.’ (Participant 12, female, specialised hospital)

‘The Department of Health then opened it up to all the other organisations, which were then involved in development.’ (Participant 9, male, JHB Metropolitan district)

‘… [*S*]ome people had more idea, like had been involved at policy level before…had policy training.’ (Participant 4, female, Central hospital)

#### Implementation of the framework and strategy for disability and rehabilitation services

The National Department of Health was responsible for implementing the FSDR together with other stakeholders.

‘We will endeavour to implement this framework and strategy to ensure that services for persons with disabilities are available at all the levels of healthcare.’ (FSDR document, p. 7)

‘Establish an Inter-sectoral Disability and Rehabilitation for Health Steering Committee to be housed within the National Health Commission.’ (FSDR document, p. 18)

The implementation process of the FSDR proved to be a challenge because of poor leadership to ensure that there is a common vision around FSDR and that it is adequately understood and implemented:

‘I think the lack of urgency, perhaps, you know, or leadership, governance, you know, of, you know, how serious do you really take the document.’ (Participant 7, female, Gauteng department of health)

‘… [*E*]verybody interprets that content in the way that they think it should and so there is not necessarily a collective vision about that, nor does it necessarily reflect the needs of the people.’ (Participant 12, female, specialised hospital)

The FSDR was not operationalised properly in terms of allocation of resources such as a dedicated budget and human resources to facilitate the implementation process:

‘I mean it [FSDR] doesn’t operationalise anything either because there aren’t resources allocated to implement it.’ (Participant 2, female, academia)

‘What was also lacking … there wasn’t an alignment to budgeting processes also. So, no commitment that, if let’s say, early intervention is a priority, what does that actually mean in terms of implementation and what does that mean in terms of provinces dedicating a budget to making it happen.’ (Participant 12, female, specialised hospital)

The lack of training resulted in some actors being unsure of the implementation process, leading to implementation differences across provinces:

‘We didn’t have any training specifically on the policy except the booklet to refer back to.’ (Participant 11, female, professional association)

‘The implementation varied from province to province.’ (Participant 8, female, National department of health)

Even though the implementation process was met with barriers, there were some facilitators to the implementation process. The national health department offered some support through mentorship training and strategic planning:

‘National department (NDOH) made itself available to take provincial actors through training, which some provinces took up and others felt they are okay.’ (Participant 9, male, JHB Metropolitan district)

‘A facilitator to help with implementation was that I think mentorship from occupational therapists in higher management posts.’ (Participant 10, female, JHB Metropolitan district)

‘… [*A*]t that stage, we were very lucky to have a company and I can’t remember its name that was doing work for Gauteng Health in terms of strategic planning, and they took that document and with senior representatives from Gauteng Health, we actually then developed a strategic plan, emanating from that.’ (Participant 8, female, National department of health)

#### Framework and strategy for disability and rehabilitation services monitoring and evaluation

Not much was mentioned in the FSDR on monitoring and evaluation except for goal six of the strategic plan 2015–2022:

‘GOAL 6: Improve monitoring and evaluation of disability and rehabilitation services develop and implement a monitoring and evaluation framework.’ (FSDR document, p. 18)

The main informant mentioned significant gaps in the monitoring and evaluation of the FSDR. There is currently no framework developed for monitoring and evaluating the implementation process of the FSDR. The participants highlighted the lack of data collection system with set indicators, baseline data and regular continually collect for monitoring the implementation:

‘I think because there’s no clear reporting structures, so we had no baseline as to what existed before the framework, there’s nothing to compare if this framework actually made any difference in terms of implementation. Indicators were not developed.’ (Participant 12, female, specialised hospital)

‘… [*I*]f you don’t know what is happening in the area, what is the burden of disease, then how can you plan any service really. So, the monitoring and the evaluation definitely needs to be addressed and so on, so that one can plan, you know, base your planning on proper evaluation and monitoring.’ (Participant 7, female, Gauteng department of health)

‘I’m not familiar with a specific strategy that was followed and so on to say, you know, you have to do it, you don’t have to do it, and by when, and so on.’ (Participant 7, female, Gauteng department of health)

A facilitator to the monitoring and evaluation process is a case study that was commissioned by the NDOH to be carried out in the KwaZulu-Natal province to evaluate the readiness to implement the FSDR:

‘The only positive … I only know that the [*non-government organisation*] was then commissioned and it probably was at the beginning of 2017 to do research on readiness for rehabilitation, to look at, I believe [*they*] wanted to know how ready South Africa is to implement this framework and strategy. And so, they took it on, and out of the nine provinces, they chose KwaZulu-Natal as the province to work in.’ (Participant 5, female, rehabilitation non government organisation)

## Discussion

A wide range of actors, representative of various sectors, expertise and experiences who were appointed through the Minister of Health were involved in the development phase of the FSDR, albeit under financial resource and time constrained conditions. Actor dynamics is key to any successful policy process (Shiffman & Smith 2007). Actor dynamics represents power (Buse et al. 2005) linked to individuals, organisational or political standing and representation (Buse et al. 2005; Shiffman & Smith 2007; Shumba & Moodley 2018). Actor dynamics is an essential part of the decisions made during policy development and influences what ends up in the agenda of any health policy document (Buse et al. 2005; Hudson, Hunter & Peckham 2019; Koduah, Van Dijk & Agyyepong 2015; Shiffman & Smith 2007). In the case of the FSDR, the key representatives of the core professional rehabilitation services providers were included, and this was evident in the FSDR, which not only considered the principles of the CRPD but also mirrored the principles of the CBR (Sherry 2014). The inclusion of Organisations for People with Disabilities (DPOs) as actors in the development of the FSDR should be acknowledged as it follows the historical pattern of inclusion of DPOs in drawing up the constitution of South Africa, as well as contributing to previous disability policy development in South Africa (Lang & Murangria 2009).

This representation of actors, which was a strength of the policy development phase, was unfortunately not maintained in subsequent stages, specifically in implementation and monitoring. Mugwagwa, Edwards and De Haan (2015:2) view policy implementation as a social action transformed from the policy, which is typically aimed at social betterment and most frequently manifests as programmes, procedures, regulations or practices. In essence, Mugwagwa et al. (2015) and the authors of this article argue that policy development and implementation require the involvement of different stakeholders, and it should not be left as a sole responsibility of the state government if it is to achieve social betterment of people at the grassroots in this case persons with disabilities, where the slogan ‘nothing for us without us’ should ring true.

Insufficient human, financial and physical resources and an unrealistic time frame allocated to the process were consistent across all policy stages and therefore significantly impeded the successful implementation of the FSDR. These were compounded by the insufficient training, skills and insufficient resource allocation for implementing the FSDR. The insufficient state government commitment to human and financial resources necessary for policy development and implementation is reconcilable with those of other developing countries and has been at the centre of debates on policy implementation gaps (Koduah et al. 2015; Masuku 2020; Mugwagwa et al. 2015; Shumba & Moodley 2018). The insufficient preparedness of staff in policy implementation skills that evoked negative attitudes and feelings towards the process is a common occurrence in disability policy studies (Masuku 2020; Mugwagwa et al. 2015; Shumba & Moodley 2018). Grindle (2017) argues that insufficient available resources contribute greatly to the variable and inconsistent nature of the policy implementation process. This insufficiency related to resource allocation, therefore, highlights the critical role and need for state governments to reflect on their commitment to the success of policy implementation through investing resources and skills training. The development of policies shouldn’t be seen only as an undertaking aimed at serving political agendas. However, there should be a realisation of the impact that insufficient implementation has on access to and satisfaction with healthcare services and rehabilitation for persons with disabilities and other vulnerable groups who depend on state facilities for their healthcare and rehabilitation needs. While the authors view that policy implementation should not be the sole responsibility of government states, the government should provide the necessary human, financial and physical resources necessary for successful policy implementation and monitoring.

The monitoring phase of any public policy aims to assess whether or not a policy or programme has achieved the objectives it set out to achieve. The FSDR’s monitoring stage was unsuccessful because of insufficient data collection systems. It has been reported that there are case studies underway to evaluate the success or failure of the policy; however, this is being performed in one province. Lamhauge, Lanzi and Agrawala (2012) corroborate that the insufficiency of a Monitoring and Evaluation framework hinders the ability of the policy actors to assess the effectiveness of the FSDR and a possible future improvement. The Hák, Janoušková and Moldan (2016) has stressed that for states to keep actively working towards achieving the Sustainable Developmental Goals, they need to respond to and prioritise policy decisions that guide public health interventions.

If policy decisions are not prioritised, be it at the development, implementation or monitoring stages, the repercussions are felt by the people at the grassroots, particularly persons with disabilities. These are evident in the barriers experienced by persons with disabilities when they access healthcare services, such as insufficient access to assistive services because of inefficient procurement systems, staff shortage, transportation challenges and poor socio-economic conditions of patients, which have been extensively documented in the local and international literature (Masuku 2020; Moodely & Ross 2015; Sherry 2014; Vergunst et al. 2017), more effort needs to be put into strengthening the implementation of policy and this can be addressed by raising awareness about the policy to the health providers and ensuring adequate resources to facilitate the implementation of the policy.

### Study limitations

This study’s findings are specific to the Johannesburg Metropolitan district on the experience of the processes of the FSDR and the barriers associated with it. The study’s findings cannot be generalised to other districts and provinces. Furthermore, the lack of end user involvement such as persons with disabilities as part of interview participants may limit the depth of the study’s findings on the barriers and facilitators to the processes of the FSDR.

## Conclusion

The FSDR has not successfully achieved the intended outcomes despite its best intentions. While the FSDR has adequately considered the principles of the CRPD and mirrors the philosophy of the CBR on which it is based, the apparent gaps in policy implementation and monitoring have significantly hindered its progress. This reaffirms the fact that South Africa has very good policies, which often lack in implementation. Therefore, this highlights the need for the country to shift its focus from developing more policies but rather investing on research that will focus on guidelines on policy implementation and strategies for policy monitoring and evaluation. Policy implementation requires multisectoral commitment, resources and key people leading the proposed reform. Very imperative to this process is the buy in from stakeholders and the general public that Lipsky (2010) terms ‘street-level bureaucrats’. A context that is appropriate and receptive to a policy reform along with positive organisational culture can contribute positively towards implementation. With specific reference to the FSDR, rehabilitation healthcare workers as well as community health workers are key to the process of implementation and should not be left behind if implementation is to be successful. The state needs to also commit resources to the process of policy implementation. While we acknowledge the role and need for multisectoral involvement, the state’s commitment is key. The National Department of Health and Gauteng Provincial Department of health need to rethink and recommit to making disability a part of their agenda and health programmes. If disability is prioritised, then policies related to disability and health will be prioritised.

## Appendix 1

**TABLE 1-A1:** Document review guide.

Theme	Variable
Theme 1: Actors in the FSDR	Identify actors in the FSDR Identify the specific roles and responsibilities of the actors
Theme 2: The context of the FSDR	Reasons for development of the FSDR Situational factors Structural factors Cultural factors Global factors and specific to SA
Theme 3: The content of the FSDR	Aims and objectives of the FSDR Assumptions Values Distributional impact Access to health for persons with disability CBR Matrix health component: promotion, prevention, medical care, rehabilitation assistive devices
Theme 4: The processes of the FSDR	Conceptualisation of the FSDR Development of the FSDR Implementation of the FSDR Evaluation of the FSDR
Interactions between the four components of the policy triangle framework	Actors at the centre of the interaction Content, context and processes interact with each other and around the actors

CBR, community-based rehabilitation; FSDR, framework and strategy for disability and rehabilitation services.

## Appendix 2

### Interview guide for semi-structured interviews

BOX 1-A2 Identification information.

**Table:**

1.1 Participant identifier (number)
1.2 Date of interview
1.3 Name of interviewer
1.4 Interview venue

BOX 2-A2 Demographic data sheet for key informants.

**Table:**

1.1 Identifier code
1.2 Gender
1.3 Age
1.4 Level of education
1.5 Occupation
1.6 Position in the department

**TABLE 1-A2:** Interview guide.

Theme	Question
Theme 1: Key informant role	Can you tell me a little bit about your role in this department? Can you tell me a little bit about your role in disability management and what your thoughts are on the FSDR? Probe: How do you feel about policy related to access to health for persons with disability?
Theme 2: The context of the FSDR	How did the FSDR come about? Probe: What was the reason for the development of the FSDR? Probe: How was the FSDR developed?
Theme 3: The content of the FSDR	How does the content of the FSDR relate to access to health for persons with disability? Probe: In your experience, what are factors affecting access to health for persons with disability?
Theme 4: The processes of the FSDR and factorsinfluencing the processes of the FSDR	In your experience, what factors affect the processes (initiation, development and implementation) of the FSDR? Probe: Did you play a role in the processes of the FSDR?
Suggestions	What are some suggestions for the processes and factors affecting the processes of the FSDR and future disability policy in South Africa? Probe: What are some suggestions to improve access to health for persons with disability?

FSDR, framework and strategy for disability and rehabilitation services.

**TABLE 2-A2:** Interview guide checklist.

Checklist	Tick
• Consent for interview and recordings	□
• Audio recorder and extra batteries	□
• Demographic information of the participant	□
• All questions addressed	□
• All responses rechecked with participant	□
